# Structural Covariance of the Prefrontal-Amygdala Pathways Associated with Heart Rate Variability

**DOI:** 10.3389/fnhum.2018.00002

**Published:** 2018-01-23

**Authors:** Luqing Wei, Hong Chen, Guo-Rong Wu

**Affiliations:** Key Laboratory of Cognition and Personality, Faculty of Psychology, Southwest University, Chongqing, China

**Keywords:** amygdala, heart rate variability, central autonomic network, structural covariance network, prefrontal-amygdala pathways

## Abstract

The neurovisceral integration model has shown a key role of the amygdala in neural circuits underlying heart rate variability (HRV) modulation, and suggested that reciprocal connections from amygdala to brain regions centered on the central autonomic network (CAN) are associated with HRV. To provide neuroanatomical evidence for these theoretical perspectives, the current study used covariance analysis of MRI-based gray matter volume (GMV) to map structural covariance network of the amygdala, and then determined whether the interregional structural correlations related to individual differences in HRV. The results showed that covariance patterns of the amygdala encompassed large portions of cortical (e.g., prefrontal, cingulate, and insula) and subcortical (e.g., striatum, hippocampus, and midbrain) regions, lending evidence from structural covariance analysis to the notion that the amygdala was a pivotal node in neural pathways for HRV modulation. Importantly, participants with higher resting HRV showed increased covariance of amygdala to dorsal medial prefrontal cortex and anterior cingulate cortex (dmPFC/dACC) extending into adjacent medial motor regions [i.e., pre-supplementary motor area (pre-SMA)/SMA], demonstrating structural covariance of the prefrontal-amygdala pathways implicated in HRV, and also implying that resting HRV may reflect the function of neural circuits underlying cognitive regulation of emotion as well as facilitation of adaptive behaviors to emotion. Our results, thus, provide anatomical substrates for the neurovisceral integration model that resting HRV may index an integrative neural network which effectively organizes emotional, cognitive, physiological and behavioral responses in the service of goal-directed behavior and adaptability.

## Introduction

Previous neuroimaging studies have advanced our understanding of the neural correlates of heart rate variability (HRV), providing convergent evidence for activation in neural structures of the central autonomic network (CAN) involved in HRV modulation (Benarroch, 1993; Thayer and Lane, 2009; Thayer et al., 2012; Beissner et al., 2013). In particular, task-evoked blood oxygenation level-dependent (BOLD) activity and regional cerebral blood flow (rCBF) changes in the medial prefrontal cortex (mPFC), anterior cingulate cortex (ACC), insula, and amygdala have shown to be tightly coupled with HRV (Lane et al., 2001; Critchley et al., 2003; Gianaros et al., 2004; Matthews et al., 2004; Neumann et al., 2006; Napadow et al., 2008; Lane et al., 2009). Despite these advances, few studies have been conducted on interregional connections between neural structures of the CAN implicated in HRV.

The neurovisceral integration model postulates that neural structures of the CAN are reciprocally connected, allowing the prefrontal cortex to exert inhibitory control over subcortical regions (e.g., amygdala, hypothalamus, and brainstem nuclei) (Thayer and Lane, 2009). HRV reflects activity of this reciprocally inhibitory cortico–subcortical neural circuit which directs multiple processes including goal-directed behavior, adaptability, and emotion and homeostatic regulation (Thayer and Lane, 2009; Thayer et al., 2012). These theoretical perspectives indicate that interregional connections between components of the CAN should be related to HRV. Recent resting state functional magnetic resonance imaging (rs-fMRI) studies using brain connectivity technique have confirmed that HRV modulation relies on functional connections between prefrontal cortical regions and amygdala (Chang et al., 2013; Sakaki et al., 2016), supporting the idea proposed by the neurovisceral integration model that the prefrontal-amygdala pathway is associated with HRV. For instance, Chang et al. (2013) found that transient increases in HRV were accompanied by transient increases in functional connectivity between amygdala and dorsal ACC (dACC). Sakaki et al. (2016) revealed that higher resting HRV was related to stronger functional connectivity between amygdala and mPFC, potentially reflecting more efficient communication between amygdala and mPFC in individuals with greater HRV. These findings demonstrate that functional connections between prefrontal cortex and amygdala covary with individual difference in baseline HRV, and also suggest that taking a network perspective on neural correlates of HRV could be better understanding of the neurovisceral integration model. However, it remains unclear whether the underlying anatomical connections between prefrontal cortex and amygdala and/or other components of the CAN (e.g., insula and midbrain) are associated with HRV. This study was designed to fill this gap by assessing interregional anatomical connections related to resting HRV.

Evidence from anatomical tracing studies indicates that the amygdala has extensive reciprocal connections with cortical (mPFC, ACC, and insula) and subcortical (e.g., hypothalamus, periaqueductal gray, and brainstem nuclei) regions (Amaral and Price, 1984; Young et al., 1994; Carmichael and Price, 1995; Ghashghaei and Barbas, 2002; Price, 2003; Ghashghaei et al., 2007). These reciprocal connections allow the amygdala to act as a pivotal node in the cortico–subcortical neural circuits that underlie HRV modulation, since it can receive direct inhibitory influence from prefrontal cortex and also have a downward influence on a wide range of subcortical regions (e.g., hypothalamus, periaqueductal gray, and brainstem nuclei) (Thayer et al., 2009; Smith et al., 2017). Hence, the present study mapped structural covariance network centered on amygdala, and hypothesized that structural correlations from amygdala to brain regions located in the reciprocally connected cortico–subcortical neural pathways would be related to HRV. Specially, structural covariance of the prefrontal-amygdala pathway was assumed to be associated with HRV, given that functional connectivity between prefrontal cortex and amygdala was correlated with HRV (Chang et al., 2013; Sakaki et al., 2016). Here we used covariance analysis of MRI-based gray matter volume (GMV) to map structural covariance network, a method that allows us to examine the pattern of covariance between the GMV of an *a priori* selected “seed” brain region and the GMV throughout the entire brain (Mechelli et al., 2005; Zielinski et al., 2010). This approach represents a valuable tool to investigate interregional structural relationship and has been successfully used in healthy brain and multiple disease state (Seeley et al., 2009; Montembeault et al., 2012, 2016; Liao et al., 2013; Spreng and Turner, 2013). Our study applied this technique to assess the relationship between amygdala-specific covariance network and individual’s HRV levels, which would contribute to our knowledge about the underlying anatomical substrates of resting HRV.

## Materials and Methods

### Data Acquisition

The MRI and physiological data acquired from the publicly available Enhanced Nathan Kline Institute-Rockland Sample (NKI-RS^1^) (Nooner et al., 2012). The NKI institutional review boards approved the data collection, and all participants gave informed consent in accordance with the Declaration of Helsinki. The participants underwent multimodal MRI scans, semi-structured diagnostic psychiatric interviews, and a battery of psychiatric, cognitive, and behavioral assessments (Nooner et al., 2012). Healthy subjects with complete demographic information and without diagnosed mental disorders were included in our study (*n* = 225, age: 18–60). Anatomical images for each subject were obtained using an MPRAGE sequence (TR/TE = 1900/2.52 ms, FA = 9°, thickness = 1.0 mm, slices = 192, matrix = 256 × 256, FOV = 250 mm). Physiological data (cardiac and respiratory recordings) were simultaneously recoded for resting state fMRI scan (TR = 0.645 s, duration 9.7 min) using a Biopac MRI compatible acquisition system (sampling rate = 62.5 Hz). The cardiac cycle was monitored using a photoplethysmographic technology.

### Data Preprocessing and HRV Analysis

The structural images were preprocessed using the SPM12 software package^2^. First, structural images were reoriented to the anterior commissure and segmented into gray matter, whiter matter, and cerebrospinal fluid, using the standard segmentation option in SPM12. Then, the images obtained in the segmentation routine were applied to create a sample-specific template using DARTEL toolbox in SPM12. The segmented gray matter images were non-linearly normalized into sample-specific template with subject-specific flow fields that were generated during template creation, and affine-aligned into MIN space (1.5 mm isometric voxel size). Finally, spatially normalized images were modulated and smoothed with an 8-mm full-width at half-maximum Gaussian kernel. In addition, HRV analysis was performed on the interbeat interval (IBI) time series using the HRV analysis software (HRVAS^3^). The IBI time series were derived from photoplethysmography signal with PhysIO Toolbox (Kasper et al., 2017). The root mean square of successive differences of the IBI series (RMSSD) was computed (TaskForce, 1996). The RMSSD is predominantly vagally mediated (Hayano et al., 1991; Hansen et al., 2003; Sztajzel, 2004) and has been used in resting state functional connectivity studies (Chang et al., 2013; Sakaki et al., 2016). The detailed procedure for HRV analysis can be found in our previous work (Wu and Marinazzo, 2016; Wei et al., 2018).

### Statistical Analysis

#### Structural Covariance Networks

Regional GMV of bilateral amygdala were extracted from the GMV images. The seed regions of bilateral amygdala were defined from the Automated Anatomical Labeling (AAL) template (Tzourio-Mazoyer et al., 2002). Two separate multiple regression models were performed on the left and right amygdala, with age, gender, and total intracranial volume (TIV) as confounding covariates. The linear model fitted for the volume *T* at a GM point *i* was (Lerch et al., 2006).

$$T_{i}=\beta_{0}+\beta_{1}\,\;T_{seed}+\beta_{2}\,\;Co\mathrm{var}iates+\epsilon$$

The statistical significance threshold was set at *p* < 0.05, corrected for multiple comparisons using family-wise error (FWE).

#### Relationship between Structural Covariance Strength and RMSSD

To assess the relationship between structural covariance strength and RMSSD, the multiple regression models included the GMV of amygdala, RMSSD, parametric interaction term between seed GMV and RMSSD, as well as confounding covariates (age, gender, and TIV). The linear model fitted for the volume *T* at a GM point *i* was (Lerch et al., 2006).

$$T_{i}=\beta_{0}+\beta_{1}\,\;T_{seed}+\beta_{2}\,\;Co\mathrm{var}iates+\beta_{3}\,\;RMSSD+\beta_{4}\left(RMSSD\times T_{seed}\right)+\epsilon$$

Where × indicated an interaction. Student’s *t*-test was conducted to test for the statistical significance of β_4_. The statistical significance threshold was set at *p* < 0.05, corrected for multiple comparisons using topological false discovery rate (topoFDR) (Chumbley et al., 2010).

## Results

### Participant Characteristics

After exclusion of six subjects with poor quality of anatomical images and 34 subjects with poor quality of photoplethysmography signal, this study contained 185 healthy subjects. **Table 1** shows the demographic characteristics as well as physiological measures (e.g., systolic blood pressure, diastolic blood pressure, heart rate, and RMSSD) for the whole sample, males, and females. No significant difference was found between males and females with regard to the physiological measures.

**Table 1:** Basic characteristics of the whole sample and subgroups.

	Whole sample *n* = 185	Males *n* = 90	Females *n* = 95	*P*-value
Age	35.19 (14.02)	31.53 (13.01)	38.66 (14.12)	<0.001
BMI	25.81 (6.79)	25.17 (7.37)	26.43 (6.15)	0.21
Systolic BP	111.30 (23.44)	112.53 (27.91)	110.11 (18.24)	0.47
Diastolic BP	71.61 (16.55)	72.12 (19.51)	71.13 (13.19)	0.68
RMSSD	39.87 (16.47)	39.63 (15.98)	40.09 (17.00)	0.85
Ln HF-HRV	6.44 (1.01)	6.41 (1.01)	6.46 (1.01)	0.75
HR	69.89 (9.52)	69.55 (9.39)	70.21 (9.69)	0.64


*Means were reported with their standard deviation in parentheses. *P*-value: males vs. females. BMI, body mass index; RMSSD, root mean square of successive differences of the IBI series; HR, heart rate; Ln HF-HRV, natural log of high-frequency heart rate variability; BP, blood pressure (mmHg).*

### Mapping Structural Covariance Networks of the Amygdala

**FIGURE 1:**
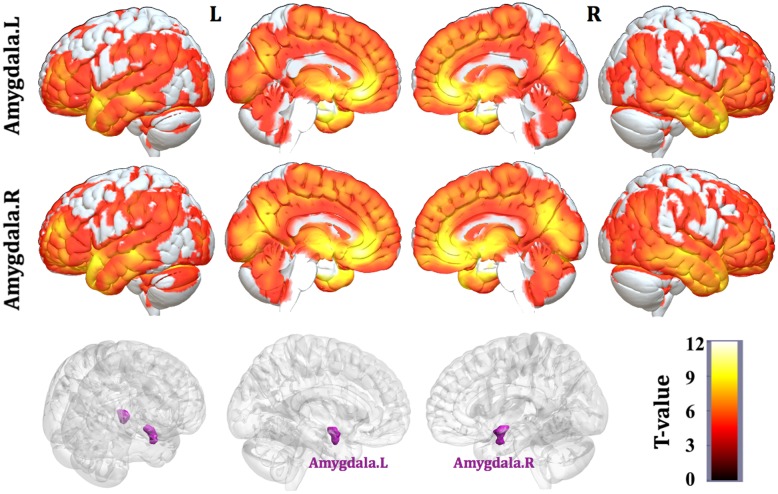
Statistical maps of structural covariance network centered on the amygdala (*p* < 0.05, FWE corrected; first row: left amygdala, second row: right amygdala, third row: seed regions). *T*-statistics are coded on a hot color scale. The first two columns of the statistical maps denote the left hemisphere, and the last two columns denote the right hemisphere.

Patterns of structural correlations with right amygdala encompassed large portions of prefrontal cortices (e.g., lateral, medial, and orbital part), sensorimotor cortices (e.g., precentral/postcentral gyrus, supplementary motor area, and paracentral lobule), cingulate cortices (e.g., ventral and dorsal anterior cingulate, midcingulate, and posterior cingulate), temporolimbic regions (e.g., insula, superior/middle/inferior temporal gyrus, temporal pole, parahippocampal gyrus, and fusiform gyrus), subcortical areas (e.g., thalamus, putamen, caudate, hippocampus, and midbrain), parietooccipital regions (e.g., inferior parietal lobule, precuneus, lingual gyrus, and cuneus), together with cerebellum (e.g., cerebellum anterior and posterior lobe) (*p* < 0.05, FWE corrected, **Table 2** and **Figure 1**). Patterns of structural correlations with left amygdala resembled the patterns observed for the right amygdala (*p* < 0.05, FWE corrected, **Figure 1** and Supplementary Table S1).

**Table 2:** Brain regions showing significant structural correlations with right amygdala.

Anatomic region	Hemisphere	BA	Peak-MNI coordinates (*x*, *y*, *z*)	Cluster size (#voxels)	*T*-value
**Cingulate cortices**			
Anterior cingulate	L	32	-2, 32, -9	2709	11.06
Anterior cingulate	R	32	2, 33, -8	2910	10.84
Midcingulate	L	31	0, -29, 50	2951	7.27
Midcingulate	R	32	8, 24, 35	3433	7.58
Posterior cingulate	R	31	5, -62, 24	2237	7.83
**Prefrontal cortices**			
Medial frontal gyrus	L	10	-9, 50, 12	4905	8.12
Medial frontal gyrus	R	32	5, 45, 0	3307	9.39
Orbital frontal gyrus	L	11	-27, 35, -15	2054	8.52
Orbital frontal gyrus	R	11	24, 33, -20	1905	7.70
Superior frontal gyrus	L	10	-17, 51, 11	2132	8.32
Superior frontal gyrus	R	9	23, 39, 41	4400	8.34
Middle frontal gyrus	L	10	-32, 50, 6	4237	8.79
Middle frontal gyrus	R	10	33, 53, 3	6206	8.36
**Sensorimotor cortices**			
Precentral gyrus	L	6	-38, 3, 18	1728	8.35
Precentral gyrus	R	6	50, -9, 42	1471	6.51
Paracentral lobule	L	31	-2, -29, 50	2234	7.29
Postcentral gyrus	L	40	-63, -23, 14	2543	8.08
Postcentral gyrus	R	6	59, -8, 33	2022	6.78
Supplementary motor area	L	6	2, 6, 50	3032	7.80
Supplementary motor area	R	6	3, 6, 48	2522	7.69
**Temporolimbic regions**			
Insula	L	13	-36, -6, -12	3936	10.75
Insula	R	13	30, 9, -18	3773	15.57
Temporal pole	L	38	-27, 3, -23	2763	18.63
Temporal pole	R	38	27, 3, -21	2600	40.95
Parahippocampal gyrus	L	34	-17, -2, -18	2064	16.68
Parahippocampal gyrus	R	34	22, 0, -21	2013	33.36
Superior temporal gyrus	L	38	-45, -3, -14	4516	11.08
Superior temporal gyrus	R	38	44, 0, -14	3868	10.70
Middle temporal gyrus	L	21	-45, 0, -17	6654	11.42
Middle temporal gyrus	R	21	56, -18, -15	5876	10.17
Inferior temporal gyrus	L	21	-36, 11, -35	4378	9.62
Inferior temporal gyrus	R	20	54, -15, -18	5457	9.64
Fusiform gyrus	L	20	-33, -6, -27	4601	11.07
Fusiform gyrus	R	20	32, 2, -32	3675	13.27
**Parietooccipital regions**			
Precuneus	L	31	-2, -66, 26	2850	8.26
Precuneus	R	31	3, -68, 21	3122	8.71
Inferior parietal lobule	L	40	-50, -56, 36	3538	6.83
Inferior parietal lobule	R	40	47, -59, 44	1066	7.12
Lingual gyrus	L	18	-10, -86, -15	2652	7.91
Lingual gyrus	R	18	17, -81, -14	2603	8.39
Cuneus	L	7	-2, -69, 21	1423	8.60
Cuneus	R	7	3, -68, 20	1465	8.65
**Subcortical regions**			
Hippocampus	L	–	-23, -6, -20	1699	18.73
Hippocampus	R	–	26, -3, -20	1475	42.69
Thalamus	R	–	6, -8, -2	1288	7.71
Thalamus	L	–	-3, -8, -2	1734	8.28
Caudate	R	–	14, 8, -12	1929	10.80
Caudate	L	–	-9, 23, -8	1698	10.36
Putamen	R	–	27, 2, -11	2208	16.50
Putamen	L	–	-20, 2, -11	1788	12.64
Midbrain	R	–	17, -9, -12	2212	15.27
Midbrain	L	-	-17, -12, -12	1422	11.60
**Cerebellum**			
Anterior and posterior lobe	R	-	21, -78, -18	7676	7.48
Anterior and posterior lobe	L	-	-26, -53, -18	9667	8.71


*R, right; L, left; BA, Brodmann area; MNI, Montreal Neuroscience Institute template.*

### Relationship between Structural Covariance Strength and RMSSD

**FIGURE 2:**
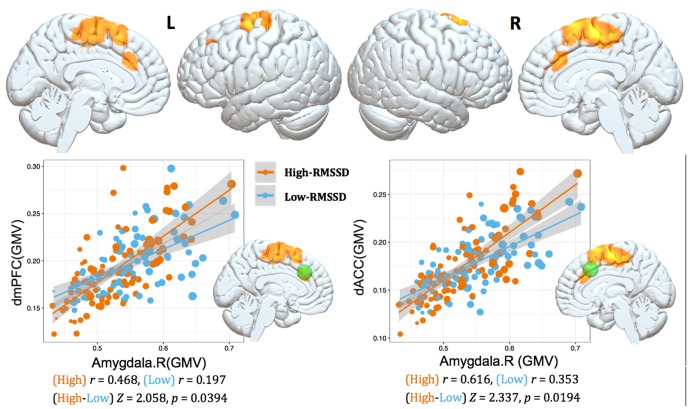
Interaction results of RMSSD and structural covariance network of the right amygdala. The first row represents positive interactions between structural covariance of the right amygdala and RMSSD (*p* < 0.05, topoFDR corrected). The second row further illustrates higher structural coupling in subjects with higher RMSSD based on two regions of interest (ROIs) analysis (Fisher’s *Z*-test, *p* < 0.05, two tail); individuals are split into high and low groups according to median value of RMSSD (i.e., RMSSD = 37.30). Gray matter volumes of the dmPFC and dACC are extracted from a sphere with 6 mm radius centered at the peak coordinate in **Table 3** (marked as green sphere). The size of scatter point is proportional to the total intracranial volume.

We studied the parametric interaction between seed covariance strength and individual differences in resting HRV, aimed to assess the relationship between structural covariance network configurations and baseline HRV levels. Structural correlations from right amygdala to dorsal mPFC/ACC (dmPFC/dACC) extending into adjacent medial motor regions (e.g., pre-supplementary motor area (pre-SMA)/SMA) were positively interacted with RMSSD (*p* < 0.05, topoFDR corrected, **Table 3** and **Figure 2**). No significant relationship was found between RMSSD and structural covariance network of the left amygdala.

**Table 3:** Relationship between structural covariance of the right amygdala seed and RMSSD.

Anatomic region	Hemisphere	BA	Peak-MNI coordinates (*x*, *y*, *z*)	Cluster Size (#voxels)	*T*-value
Pre-SMA/SMA	R	6	3, -11, 60	1950	3.52
Superior frontal gyrus	L	8	-24, -6, 68	1549	3.21
dmPFC	L	9	-15, 30, 32	748	3.00
dACC	R	32	17, 21, 39	430	3.32


*SMA, supplementary motor area; dmPFC, dorsomedial prefrontal cortex; dACC, dorsal anterior cingulate cortex; R, right; L, left; BA, Brodmann area; MNI, Montreal Neuroscience Institute template.*

To further illustrate the parametric interaction effect, partial correlation analysis (with gender, age and TIV as confounding covariates) between amygdala GMV and target region’s GMV was performed after the group has been split into two groups (91 vs. 94 subjects) according to the median value of RMSSD (i.e., RMSSD = 37.30). The significance of the differences between correlation coefficients in two groups was determined by Fisher’s *Z*-test (Meng et al., 1992). The results showed that individuals with higher RMSSD values exhibited stronger structural correlations between right amygdala and dmPFC/dACC relative to those with lower RMSSD values (Fisher’s *Z*-test, *p* < 0.05, **Figure 2**), further indicating significant parametric interaction effect between covariance strength and HRV levels.

### Relationship between Structural Covariance Strength and Heart Rate

**FIGURE 3:**
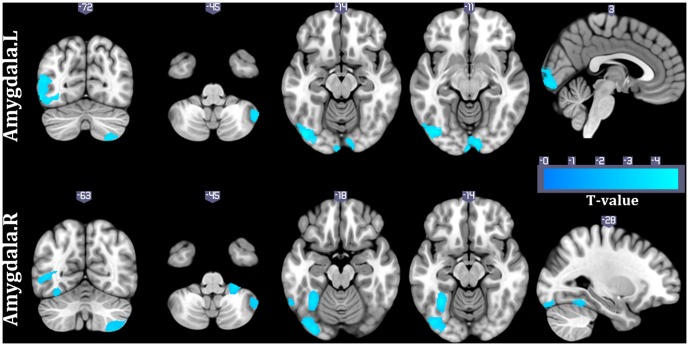
Interaction results of HR and structural covariance network of the amygdala (*p* < 0.05, topoFDR corrected).

To validate whether the associations between HRV and structural covariance strength were confounded by heart rate (HR), we also examined the relationship between structural covariance network of amygdala and HR. Structural correlations from right amygdala to right cerebellum, left middle temporal gyrus and left middle occipital gyrus were negatively interacted with HR (*p* < 0.05, topoFDR corrected, **Figure 3**). Similar results were obtained for the left amygdala seed region (*p* < 0.05, topoFDR corrected, **Figure 3**). These results validated that structural covariance from prefrontal cortical regions (e.g., dmPFC, dACC, and pre-SMA) to amygdala was associated with changes in HRV rather than changes in HR.

## Discussion

Based on covariance analysis of structural MRI-based GMV measurements, we assessed the relationship between structural brain network of amygdala and individual differences in resting HRV. To best of our knowledge, this is the first study to investigate the interregional structural correlations associated with individual’s HRV levels. The results showed widespread structural correlations of amygdala to cortical and subcortical regions, extending previous tract tracing and functional connectivity results to the domain of interregional structural covariance patterns in the brain, suggesting that the amygdala serves as a crucial node in neural circuits underlying HRV regulation (Thayer and Lane, 2009; Thayer et al., 2012). More importantly, structural covariance of amygdala to dmPFC/dACC and adjacent pre-SMA/SMA was related to baseline HRV, providing anatomical evidence for the neurovisceral integration model that the prefrontal-amygdala pathways contribute to individual differences in resting HRV.

Complementary to fMRI and diffusion tensor imaging (DTI) based connectomics, structural MRI-based covariance analysis represents another source of information about interregional anatomical associations. Characterization of such a network is crucial for revealing intrinsically structural organizational principles in human brain and enhancing our understanding of how functional brain states emerge from their underlying structural substrates (He et al., 2007). The covariation of brain morphology (e.g., cortical thickness and GMV) in related regions was possibly resulted from mutual trophic influences (i.e., changes in axonal connections or blood supply) (Ferrer et al., 1995), genetic influences (Schmitt et al., 2008), or common experience-related plasticity (Draganski et al., 2004; Mechelli et al., 2004). The pattern of structural covariance was associated with the pattern of functional and/or white matter (WM) connectivity (He et al., 2007; Seeley et al., 2009), and areas that covary in morphological characteristics may be part of the same functional networks (Montembeault et al., 2012, 2016). In the current study, structural covariance network of the amygdala was widespread and resembled findings from anatomical tract tracing studies, showing amygdala interconnections with multiple prefrontal, cingulate, sensorimotor, temporolimbic, and subcortical regions (Amaral and Price, 1984; Young et al., 1994; Ghashghaei and Barbas, 2002; Price, 2003; Swanson, 2003). Moreover, structural covariance patterns of amygdala in our study were similar to functional connectivity patterns of amygdala (Stein et al., 2007; Roy et al., 2009; Robinson et al., 2010). The current findings, thus, lend evidence from structural covariance analysis to the notion that the amygdala reciprocally connected with cortical (e.g., prefrontal, cingulate, and insular cortices) and subcortical (e.g., striatum, hippocampus, and midbrain) regions is a pivotal node in neural circuits for HRV regulation (Thayer and Lane, 2009; Thayer et al., 2012).

Our main goal was sought to determine whether structural covariance network centered on the amygdala associated with individual difference in resting HRV. This was accomplished by discovering structural covariance of amygdala to dmPFC/dACC related to resting HRV. The dmPFC and dACC have direct projections to subcortical brain regions associated with homeostasis and autonomic control, such as the amygdala, periaqueductal gray (PAG), hypothalamus and pons (Vilensky and van Hoesen, 1981; An et al., 1998; Ongur et al., 1998; Ghashghaei et al., 2007). These anatomical connections support the role of the dmPFC and dACC in autonomic function. Investigators using task-evoked fMRI, PET and arterial spin labeling (ASL) perfusion technique have demonstrated dmPFC and dACC activation correlated with HRV changes (Lane et al., 2001; Critchley et al., 2003; Neumann et al., 2006; Napadow et al., 2008; Kano et al., 2014). For example, Neumann et al. (2006) found an inverse association between baseline HRV and dACC activity during a go/no-go task. Critchley et al. (2003) discovered dACC activity associated with HRV changes during performance of cognitive and motor tasks. With respect to dmPFC, a positive association was observed between dmPFC activation and HRV during emotion-laden and handgrip task (Lane et al., 2001; Napadow et al., 2008). In addition, a meta-analysis of human emotion studies indicated that activation in dmPFC was often co-activated with regions known to influence autonomic physiology (i.e., PAG and hypothalamus), suggesting that dmPFC activation during reappraisal-based emotion regulation should be associated with changes in autonomic physiology (Kober et al., 2008). Taken together, the above-mentioned evidence has confirmed the involvement of the dACC and dmPFC in regulation of HRV.

The neurovisceral integration model proposes that inhibitory processes from prefrontal cortex to amygdala are crucial for HRV modulation, indicating the prefrontal-amygdala pathways implicated in HRV (Thayer and Lane, 2009; Thayer et al., 2012). Previous neuroimaging studies have linked functional couplings of the prefrontal-amygdala circuits with cardiovascular arousal (e.g., HRV and blood pressure reactivity) (Gianaros et al., 2008; Chang et al., 2013; Sakaki et al., 2016). The current results extend these findings by showing that inter-regional covariance patterns of dmPFC/dACC and amygdala coupled with HRV, demonstrating the anatomical covariance of the prefrontal-amygdala network associated with resting HRV, and also suggesting that inter-regional covariance analysis in the structural domain opens a new avenue to understand the neural mechanisms for HRV regulation. Besides, it is worth pointing out that the dmPFC/dACC-amygdala pathways we found here have traditionally been regarded as important neural circuits for cognitive regulation of emotion (Ochsner et al., 2004; Quirk and Beer, 2006; Ochsner and Gross, 2008; Etkin et al., 2011). The dmPFC and dACC were consistently activated during reappraisal/suppression-based affect regulation (Ochsner et al., 2002; Levesque et al., 2003; Banks et al., 2007; Buhle et al., 2014). Previous neuroimaging studies investigated the importance of dmPFC/dACC-amygdala circuits in the context of affect regulation by assessing the inter-regional relationship between dmPFC/dACC and amygdala during cognitive-emotional tasks (Phan et al., 2005; Urry et al., 2006; Banks et al., 2007), demonstrating that stronger functional coupling exists between amygdala and dmPFC/dACC for the reduction or down-regulation of negative emotion (Pezawas et al., 2005; Urry et al., 2006; Banks et al., 2007; Kim et al., 2011). Based on the well-known relationship between resting HRV and emotion regulation (Thayer and Lane, 2000; Butler et al., 2006; Williams et al., 2015), the current findings may support that higher resting HRV was associated with neural mechanisms underlying successful emotion regulation (Thayer and Lane, 2009; Thayer et al., 2012; Sakaki et al., 2016). As the relationship among resting HRV, emotion regulation and the dmPFC/dACC-amygdala circuit was not verified in current study, further validation of the statement that emotion regulation and autonomic control shared neural substrates in the prefrontal-amygdala pathway is needed.

Finally, structural correlation between amygdala and pre-SMA/SMA was related to baseline HRV. The pre-SMA/SMA, according to a recent literature, was directly connected to sympathetic effector organs (e.g., adrenal medulla) and was potential sources of central commands to influence sympathetic arousal (Dum et al., 2016). This conclusion was also supported by a classic study that surface stimulation in medial cortical motor regions evoked changes in blood pressure (Wall and Pribram, 1950). The above findings underscore the importance of pre-SMA/SMA in regulation of autonomic states. Evidence from animal research indicated that the pre-SMA/SMA received direct projections from the amygdala (Macchi et al., 1978; Jurgens, 1984; Sripanidkulchai et al., 1984). In humans, diffusion tensor imaging and resting-state fMRI technique have identified direct anatomical and functional connections between amygdala and pre-SMA/SMA (Grezes et al., 2014; Toschi et al., 2017). The direct link between amygdala and pre-SMA/SMA suggest that the amygdala may work in tandem with cortical motor areas to facilitate the preparation of adaptive behavioral responses to affective signals (Grezes et al., 2014; Toschi et al., 2017). This is also consistent with previous task-based fMRI findings which found co-activation of the amygdala and cortical motor areas during emotional processing (de Gelder et al., 2004; Ahs et al., 2009; Conty et al., 2012; Grezes et al., 2013; Kragel and LaBar, 2016), and with evidence from transcranial magnetic stimulation studies showing that emotional stimuli prime the motor system and facilitate action readiness (Oliveri et al., 2003; Hajcak et al., 2007; Coombes et al., 2009; Coelho et al., 2010). On the basis of the viewpoint that interactions between amygdala and cortical motor systems occur to mediate adaptive behaviors to affective stimuli (Grezes et al., 2014; Toschi et al., 2017), the current results may suggest that resting HRV was associated with the neural pathway important for facilitation of adaptive behaviors to emotion (Thayer and Lane, 2009; Thayer et al., 2012; Gillie and Thayer, 2014).

In summary, our findings showed that structural covariance network of the amygdala was centered on the CAN (e.g., prefrontal, cingulate, insula, striatum, hippocampus, and midbrain), confirming the amygdala plays a key role in neural circuits for HRV modulation. Moreover, structural covariance between amygdala and dmPFC/dACC encompassing adjacent pre-SMA/SMA were related to individual differences in HRV, demonstrating the prefrontal-amygdala pathways involved in resting HRV. The current results link neural circuits accounting for cognitive control of emotion and facilitation of adaptive behavior to emotion with the function of HRV, supporting the idea, proposed by the neurovisceral integration model, that resting HRV may index an integrative neural network which flexibly regulates emotional, cognitive, physiological and behavioral responses in the service of goal-directed behavior and adaptation. Our structural covariance findings, thus, might open a new methodological window to investigate the neural mechanisms for HRV. Additionally, high HRV is associated with low cortisol level, indicating that there is a balance between the autonomic nervous system (ANS) and the hypothalamic-pituitary-adrenal (HPA) axis in healthy subjects (Pellissier et al., 2014). However, this balance was not observed in Crohn’s disease (CD) and irritable bowel syndrome (IBS) patients, probably due to dysfunction of the prefrontal-amygdala pathways in those diseases (Pellissier et al., 2010, 2014). Our structural covariance findings might be helpful for understanding the imbalanced homeostatic regulation in pathological conditions.

## Ethics Statement

This study was carried out in accordance with the recommendations of the ethical standards of the NKI institutional review boards with written informed consent from all subjects. All subjects gave written informed consent in accordance with the Declaration of Helsinki. The protocol was approved by the NKI Institutional Review Boards.

## Author Contributions

HC and G-RW contributed to study design. LW and G-RW contributed to data collection and analysis. LW, HC, and G-RW contributed to literature review and writing of the manuscript.

## Conflict of Interest Statement

The authors declare that the research was conducted in the absence of any commercial or financial relationships that could be construed as a potential conflict of interest.

## Footnotes

1 http://fcon_1000.projects.nitrc.org/indi/enhanced/

2 http://www.fil.ion.ucl.ac.uk/spm

3 https://github.com/jramshur/HRVAS
