# Supplementary material for: Structural Covariance of the Prefrontal-Amygdala Pathways Associated with Heart Rate Variability
Source: Front Hum Neurosci. 2018 Jan 23;12:2. doi: 10.3389/fnhum.2018.00002 (PMC5838315; doi:10.3389/fnhum.2018.00002)
Supplement: Supplementary file 1 [file Table_1.docx]

Supplementary Tables

Supplementary Table S1. Brain regions showing significant structural correlations with left amygdala

| Anatomic region | Hemisphere | BA | Peak-MNI  coordinates  (x, y, z) | Cluster Size  (#voxels) | T value |
| --- | --- | --- | --- | --- | --- |
| **cingulate cortices** | | | | | |
| anterior cingulate | L | 32 | -2, 30, -9 | 2607 | 10.40 |
| anterior cingulate | R | 32 | 2, 33, -8 | 3032 | 10.28 |
| midcingulate | L | 31 | 0, -29, 50 | 3159 | 7.62 |
| midcingulate | R | 32 | 2, -26, 48 | 4001 | 7.56 |
| **prefrontal cortices** | | | | | |
| medial frontal gyrus | L | 10 | -8, 48, 6 | 5364 | 7.82 |
| medial frontal gyrus | R | 32 | 2, 45, 0 | 3541 | 9.14 |
| orbital frontal gyrus | L | 11 | -29, 33, -17 | 2091 | 8.85 |
| orbital frontal gyrus | R | 11 | 30, 53, -2 | 1999 | 7.97 |
| superior frontal gyrus | L | 10 | -17, 53, 12 | 3233 | 8.46 |
| superior frontal gyrus | R | 9 | 23, 51, 23 | 4371 | 8.02 |
| middle frontal gyrus | L | 10 | -29, 59, 11 | 5284 | 8.72 |
| middle frontal gyrus | R | 10 | 32, 54, 2 | 5774 | 8.57 |
| **sensorimotor cortices** | | | | | |
| precentral gyrus | L | 6 | -38, 3, 18 | 2670 | 7.68 |
| precentral gyrus | R | 3 | 23, -32, 60 | 2185 | 6.84 |
| paracentral lobule | L | 31 | -2, -29, 50 | 1788 | 7.49 |
| paracentral lobule | R | 5 | 3, -33, 51 | 1023 | 6.66 |
| postcentral gyrus | L | 40 | -63, -23, 14 | 3507 | 7.96 |
| postcentral gyrus | R | 6 | 27, -30, 60 | 3071 | 7.11 |
| supplementary motor area | L | 6 | 0, 8, 53 | 3074 | 7.55 |
| supplementary motor area | R | 6 | 3, 6, 48 | 2771 | 7.40 |
| **temporolimbic regions** | | | | | |
| insula | L | 13 | -26, 8, -18 | 4017 | 12.57 |
| insula | R | 13 | 29, 9, -18 | 3898 | 12.50 |
| temporal pole | L | 38 | -26, 3, -21 | 2799 | 34.04 |
| temporal pole | R | 38 | 26, 3, -21 | 2623 | 20.38 |
| parahippocampa gyrus | L | 34 | -17, -2, -18 | 2133 | 31.29 |
| parahippocampa gyrus | R | 34 | 23, 0, -21 | 2005 | 21.08 |
| superior temporal gyrus | L | 38 | -45, -3, -14 | 4247 | 11.07 |
| superior temporal gyrus | R | 38 | 44, -11, -9 | 4417 | 10.14 |
| middle temporal gyrus | L | 21 | -39, -2, -27 | 6454 | 11.57 |
| middle temporal gyrus | R | 21 | 60, -14, -11 | 6183 | 9.81 |
| inferior temporal gyrus | L | 21 | -36, 11, -33 | 4450 | 9.99 |
| inferior temporal gyrus | R | 20 | 54, -15, -18 | 5443 | 8.97 |
| fusiform gyrus | L | 20 | -32, -9, -26 | 4829 | 13.35 |
| fusiform gyrus | R | 20 | 33, -2, -30 | 4071 | 11.21 |
| **parietooccipital regions** | | | | | |
| precuneus | R | 31 | 2, -68, 23 | 2185 | 9.36 |
| precuneus | L | 31 | -2, -66, 26 | 4174 | 9.23 |
| inferior parietal lobule | L | 40 | -29, -42, 47 | 4136 | 7.91 |
| inferior parietal lobule | R | 40 | 51, -59, 39 | 1519 | 6.87 |
| lingual gyrus | L | 18 | -12, -84, -14 | 3712 | 8.80 |
| lingual gyrus | R | 18 | 18, -75, -12 | 3417 | 9.52 |
| cuneus | L | 7 | -2, -68, 24 | 1951 | 9.36 |
| cuneus | R | 7 | 3, -66, 20 | 1819 | 9.09 |
| **subcortical regions** | | | | | |
| hippocampus | L | - | -23, -6, -20 | 1802 | 41.02 |
| hippocampus | R | - | 23, -2, -20 | 1475 | 21.87 |
| thalamus | R | - | 6, -8, -2 | 1649 | 8.51 |
| thalamus | L | - | -3, -8, -2 | 1907 | 9.40 |
| caudate | R | - | 6, 6, -6 | 1870 | 9.81 |
| caudate | L | - | -9, 23, -6 | 1617 | 10.89 |
| putamen | R | - | 27, 2, -11 | 2176 | 12.12 |
| putamen | L | - | -24, 0, -11 | 1835 | 14.52 |
| midbrain | R | - | 17, -9, -12 | 1501 | 12.31 |
| midbrain | L | - | -17, -12, -12 | 1458 | 13.11 |
| **cerebellum** | | | | | |
| anterior and posterior lobe | R | - | 18, -71, -15 | 10284 | 8.32 |
| anterior and posterior lobe | L | - | -26, -56, -17 | 12555 | 9.67 |

Abbreviations: R, right; L, left; BA, Brodman area; MNI, Montreal Neuroscience Institute template.
